# Oridonin Sensitizes Cisplatin-Induced Apoptosis via AMPK/Akt/mTOR-Dependent Autophagosome Accumulation in A549 Cells

**DOI:** 10.3389/fonc.2019.00769

**Published:** 2019-08-14

**Authors:** Huahong Yang, Yun Gao, Xiaoye Fan, Xingkai Liu, Liping Peng, Xinxin Ci

**Affiliations:** ^1^Department of Respiratory Medicine, The First Hospital of Jilin University, Changchun, China; ^2^Institute of Translational Medicine, The First Hospital of Jilin University, Changchun, China; ^3^Department of Hepatopancreatobiliary Surgery, The First Hospital of Jilin University, Changchun, China

**Keywords:** oridonin, non-small cell lung cancer, cisplatin sensitivity, autophagy, apoptosis, AMPK/Akt/mTOR

## Abstract

AMPK-mediated autophagy and Akt/mTOR pathways play important roles in current cancer treatments. Oridonin (Ori), an ent-kaurane diterpenoid isolated from Isodon rubescens, exerts extensive anti-tumor potential and controversial effects on autophagy. In this study, we investigated the effect of Ori on the autophagy, apoptosis, and AMPK/Akt/mTOR pathways and determined whether Ori was related to the increased cisplatin sensitivity observed in A549 cells. First, we found that Ori suppressed Akt/mTOR, Bcl2 and autophagy flux with enhanced levels of Atg3, P62, and LC3II, which was also shown as the accumulation of autophagosomes. AMPK and pro-apoptotic proteins (caspase3, Bax, and PARP) were activated in Ori-treated cells. With the pretreatment of compound c (AMPK inhibitor), the activation of autophagosomes, apoptosis and the inhibition of Akt/mTOR pathways induced by Ori were all reversed. The Ori-activated apoptosis-related markers mentioned previously and the cell-killing effect were restrained by 3-MA (inhibitor of autophagosomes) treatment. Therefore, we hypothesized that the Ori-induced pro-apoptotic effect was mediated by AMPK/Akt/mTOR-dependent accumulation of impaired autophagosomes. Furthermore, Ori could increase the sensitivity of cisplatin through its increased cell-killing, autophagy-suppressing and apoptosis-inducing activities. In addition to sensitizing cisplatin, Ori also alleviated cisplatin-induced acute renal injury *in vivo*, manifested as depleted BUN, CRE, kidney index, and weight loss compared to the cisplatin group. In summary, apart from its protective effect on cisplatin-induced nephrotoxicity, Ori enhanced cisplatin sensitivity via its pro-apoptotic activity mediated by AMPK/Akt/mTOR-dependent autophagosome activation, which may be a potential therapeutic target for non-small cell lung cancer.

## Introduction

With the highest morbidity and mortality, lung cancer is a serious disease affecting public health (1). In terms of biological characteristics, lung cancer is divided into small cell lung cancer (SCLC) and non-small cell lung cancer (NSCLC). NSCLC accounts for 80–85% of all lung cancers with poor prognosis (2). Cisplatin remains the first-line chemotherapy drug for NSCLC, despite the occurrence of cisplatin resistance. Numerous studies show that some cytotoxic agents can reverse the cisplatin resistance of NSCLC patients, however, the survival rates are still low (3–5). Therefore, there is an urgent need to develop new effective adjuvant therapies against NSCLC.

Autophagy and relevant pathways are closely related to the emergence of cisplatin resistance (6). Autophagy, also known as “self-eating,” is the process of transporting damaged, denatured or aged proteins and organelles to lysosomes for digestion and degradation. Autophagy consists of three stages: formation of autophagosomes, fusion of autophagosome-lysosome and degradation of autophagolysosomes. There is growing evidence that autophagy takes part in many human diseases, such as cancer, neurodegenerative disease, metabolic diseases and immune-related disorders (7–9). From the studies of autophagy and cancer, autophagy takes part in tumorigenesis, metastasis and cisplatin resistance (10). Cisplatin resistance can be induced by the activation of autophagy, and the autophagy inhibitor chloroquine (CQ) can improve the sensitivity of endometrial cancer cells to cisplatin (11, 12). CQ blocks the fusion of autophagosomes and lysosomes and causes the accumulation of autophagosomes, which highlights the relationship between autophagosomes and cisplatin resistance. Moreover, recent studies show that the activation of autophagosomes inhibits the proliferation of tumor cells and sensitizes cisplatin-induced resistance in cervical cancer cells (13, 14). Therefore, finding a compound that induces autophagosome accumulation may be a new measure to reverse chemotherapy resistance.

Many signaling pathways are involved in the regulation of autophagy, such as AMPK and Akt/mTOR (15, 16). The mammalian target of rapamycin (mTOR) acts as a negative regulator of autophagy and exists in two complexes (mTORC1 and mTORC2). Protein kinase B (Akt) and AMP-activated protein kinase (AMPK) pathways can regulate mTORC1 positively and negatively (17). Previous studies have shown that Akt inhibition significantly reduces the phosphorylation of mTOR and enhances cell autophagy in human oral cancer CAR cells (18). Additionally, AMPK inhibits mTORC1 through direct phosphorylation of the tumor suppressor TSC2 on Ser 1387 or subunit Raptor on two conserved serines and subsequently triggers autophagy flux (17). In addition, targeting AMPK and Akt/mTOR signaling can overcome cisplatin resistance in ovarian and oral cancer cells, respectively (18, 19).

As a vital effector of autophagy regulation, apoptosis can be initiated to counteract the proliferation, metastasis, and cisplatin resistance of cancer cells, so that many activators of apoptosis are used in cancer treatment (20). Studies have demonstrated that 3-MA (autophagy inhibitor) increased cisplatin-induced apoptosis by increasing endoplasmic reticulum stress in U251 human glioma cells (21). Unfolded protein response suppresses cisplatin-induced apoptosis via autophagy regulation in human hepatocellular carcinoma cells (22). Therefore, in our study, we mainly studied the role of above pathways in cisplatin resistance in NSCLC cells.

An ent-kaurane diterpenoid isolated from Isodon rubescens, Oridonin (Ori) and its analogs exert anti-tumor potential in cancer cells (23–25). Besides, the mechanisms involved are mainly concentrated on autophagy and apoptosis (26, 27). However, the effect of Ori on autophagy in cisplatin-induced lung cancer cells has not been thoroughly elucidated to date. It has been verified that Ori could induce the conversion of LC3II/I in A549 cells, but the fusion of autophagosome-lysosome and the expression of P62 (marker of autophagy degradation) have not been investigated (27). So in our study, we aimed to explore the role and correlation of autophagy, apoptosis and AMPK/Akt/mTOR induced by Ori in cisplatin-treated A549 cells, and provided a new therapeutic target against carcinogenesis and cisplatin resistance in lung cancer.

## Materials and Methods

### Reagents

Oridonin was obtained from Chengdu Pufeide Biotechnology Company. All of the primary antibodies LC3, P62, Atg3, Bax, Bcl2, caspase3, PARP, P-AMPK, AMPK, P-Akt, Akt, P-mTOR, mTOR, and β-actin were from Cell Signaling Technology or Abcam. FITC-annexin V and propidium iodide (PI) were from BD or Invitrogen. Cell culture medium DMEM, antibiotic-antimycotic and trypsin–EDTA were from Corning, MBI and Biofil, respectively. Fetal bovine serum (FBS) and PBS were obtained from BI. Autophagy detection kit was obtained from Abcam. Cell Counting Kit-8 (CCK-8) was purchased from Bimake. BUN and CRE detection kits were obtained from Nanjing jiancheng Biotechnology Company. BCA protein assay kit was provided from Thermo.

### Animals

C57BL/6 WT mice were obtained from Liaoning Changsheng Technology Industrial, Co., Ltd. (Certificate SCXK2010-0001; Liaoning, China). All mice were kept in SPF-grade animal room and fed with sterile water and standard rodent chow. All animal studies were reviewed and approved by the Animal Welfare and Research Ethics Committee of Jilin University.

### Cell Culture

A549 (human NSCLC cell) and B2b (human bronchial epithelium cell) were purchased from the China Cell Line Bank (Beijing, China). Cells were maintained in DMEM supplemented with 10% FBS, 1% antibiotic-antimycotic and incubated at 37°C in a 5% CO_2_ atmosphere.

### Cell Viability Assay

A549 and B2b cells were seeded in 96-well plates (1.5 × 10^4^ cells/well) and treated with different doses of Ori or cisplatin for 18 h. Then 10 μl CCK-8 was added to every well for 1 h and measured at 450 nm according to the instructions.

### Determination of Combination Index

The interaction between Ori and cisplatin was determined by the combination index (CI) according to Chou-Talalay's median-effect plots and isobologram principles (28). Cells were treated with different doses of each single drug or their combinations at the set molar ratios (1:1). The equation for the isobologram was shown as CI = (D)1/(Dx)1 + (D)2/(Dx)2. (Dx)1 and (Dx)2 indicated the individual doses of Ori and cisplatin required to inhibit a given level of cell viability, and (D)1 and (D)2 were the doses of them necessary to produce the same effect in combination. The combination effects were indicated as follows: CI = 1, additive effect; CI > 1, antagonism; CI < 1, synergism.

### Flow Cytometry (FCM) Analysis of Apoptosis

After 18 h of drug treatment (20 μM Ori and 20 μM cisplatin), the cells were detached from the culture plates, washed with PBS and suspended in 5 μl of Annexin V binding buffer for 20 min in the dark. Then, the cells were stained with 2 μl of PI and analyzed with a FACS.

### Quantification of Autophagosomes

This step was performed using autophagy detection kit (Abcam 139484). Cells were seeded in 96-well plates and treated with different reagents (20 μM Ori, 20 μM cisplatin and 5 μM compound c) for 18 h. Starvation and CQ (50 μM) were positive controls. Then, the cells were stained with dual detection reagent and incubated for 30 min at 37°C. After washed with assay buffer, the cells were fixed with 4% formaldehyde. Finally, images were captured with a fluorescence microscope and the fluorescence intensity of autophagosomes (green) was examined by Image J.

### Immunofluorescence Staining

Cells were seeded in glassy plates and treated with Ori (20 μM), cisplatin (20 μM) or compound c (5 μM) for 18 h. After fixation in 4% paraformaldehyde for 30 min, cells were permeabilized with 0.2% Triton X-100 for 10 min and blocked with goat serum. Then, cells were incubated with primary antibody (anti-LC3) overnight and the corresponding secondary antibody. The nuclei were stained with DAPI for 20 min and images were captured with a confocal microscope. LC3 puncta were green and localized in the cytoplasm and nuclei were stained blue. Then the intensity of LC3 puncta was examined by Image J.

### Acridine Orange Staining

A549 cells (1 × 10^6^ per well) were treated with Ori (20 μM) and CQ (50 μM) for 18 h and incubated with acridine orange solution for 15 min and images were collected by fluorescence microscope.

### Western Blot

Cells were homogenized in RIPA lysis buffer that contained protease and phosphatase inhibitors. Protein concentrations were determined using a BCA protein assay kit (Thermo 23227). According to the instructions, reagent A and B were mixed together in a ratio of 50 to 1 and added into every well for the measurement of OD. Then proteins were quantified and separated by SDS-PAGE (10–12.5%) and transferred to PVDF membrane. Then blocked with non-fat milk for 1 h and incubated with primary antibodies overnight. The next day, the membrane were washed and followed with secondary antibodies for 1 h and detected by ECL.

### Experimental Models of AKI and Biochemical Assay

The mice were fasted for 12 h and randomly divided into 4 groups: the control group without any treatment (Con, *n* = 5); cisplatin-treated group (20 mg/kg, Cis, *n* = 5); Ori-treated group without cisplatin (20 mg/kg, Ori, *n* = 5); Ori-treated group with cisplatin treatment (Ori+Cis, *n* = 5). Cisplatin (20 mg/kg) was injected for 72 h to construct an acute kidney injury model, and 20 mg/kg Ori was given for 3 consecutive days. Mice were sacrificed at 24 h after the last Ori administration and body and kidney weights were recorded. Blood was collected to test BUN and CRE levels according to the manufacturer's instructions.

### Statistical Analysis

Data presented are representatives from at least three independent experiments. All results in this study were expressed as the means ± SEM and analyzed using SPSS19.0 (IBM). Statistical analysis was employed the unpaired Student's *t*-test by using GraphPad 5 Software. Value of *p* > 0.05 was considered significant.

## Results

### Ori Regulates Autophagy, AMPK, Akt/mTOR Signaling, and Apoptosis

**Figure 1:**
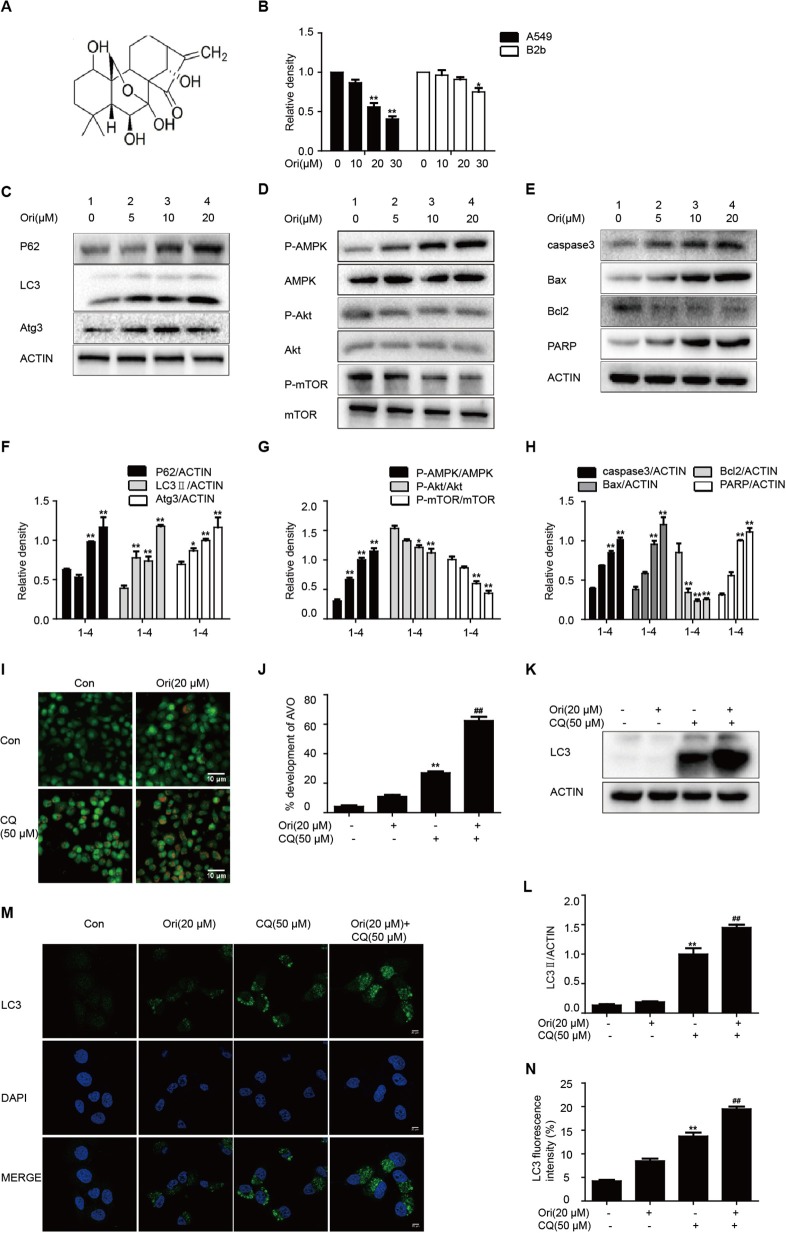
Ori regulates autophagy, AMPK, Akt/mTOR signaling and apoptosis. **(A)** The structure of Oridonin (Ori). **(B)** A549 cells and B2b cells were treated with different doses (10, 20, and 30 μM) of Ori for 18 h. Cell viability was calculated as the percentage relative to untreated cells which were considered as 100%. **(C–H)** Cells were treated with different doses of Ori (5, 10, and 20 μM) for 18 h. Representative western blots and statistical results showed the protein levels of P62, LC3, Atg3, P-AMPK, AMPK, P-Akt, Akt, P-mTOR, mTOR, caspase3, Bax, Bcl2, and PARP. **(I,J)** Cells were treated with Ori (20 μM) in the absence or presence of CQ (50 μM) for 18 h. Acidic vesicular organelles (autophagosomes) were stained with acridine orange. Scale bars: 10 μM. **(K–N)** Cells were treated with Ori (20 μM) in the absence or presence of CQ (50 μM) for 18 h. LC3 was measured with immunoblot and immunofluorescence. Scale bars: 20 μM. The results showed the average of three independent experiments. *p > 0.05 and **p > 0.01 vs. the control group. ^*##*^*p* > 0.01 vs. the CQ group.

First, we treated cells with different doses of Ori (10, 20, and 30 μM), and found that Ori could significantly inhibit the viability of A549 cells in a dose-dependent manner. Then the same doses were used in B2b cells, and we ultimately chose Ori (5, 10, and 20 μM) for the following vitro experiments (Figure 1B). As autophagy and related pathways play a vital role in cancer treatment, we investigated whether Ori had the corresponding regulatory effects in A549 cells. Autophagy-related proteins (P62, LC3, and Atg3) were activated in a dose-dependent manner (Figures 1C,F). We subsequently observed that Ori (10 and 20 μM) evidently activated AMPK, caspase3, Bax, and PARP, but decreased the protein levels of Akt, mTOR, and Bcl2 (Figures 1D,E,G,H). From our results, 20 μM Ori showed the most obvious regulator effects. These results indicate that Ori can regulate autophagy, AMPK, Akt/mTOR signaling and apoptosis in A549 cells. From the results that P62 and LC3 were all overexpressed in Ori-treated cells, we added CQ (50 μM) through acridine orange staining, LC3 staining and Western blotting to explore whether Ori inhibited autophagy flux. Results showed that Ori (20 μM) induced more acidic impaired autophagosomes and LC3 expression in CQ-treated cells (Figures 1I–N), which indicated that Ori, similar to CQ, could block the maturation and degradation of autophagosomes.

### Ori Initiates Autophagy, Apoptosis, and Akt/mTOR Signaling by the Regulation of AMPK

**Figure 2:**
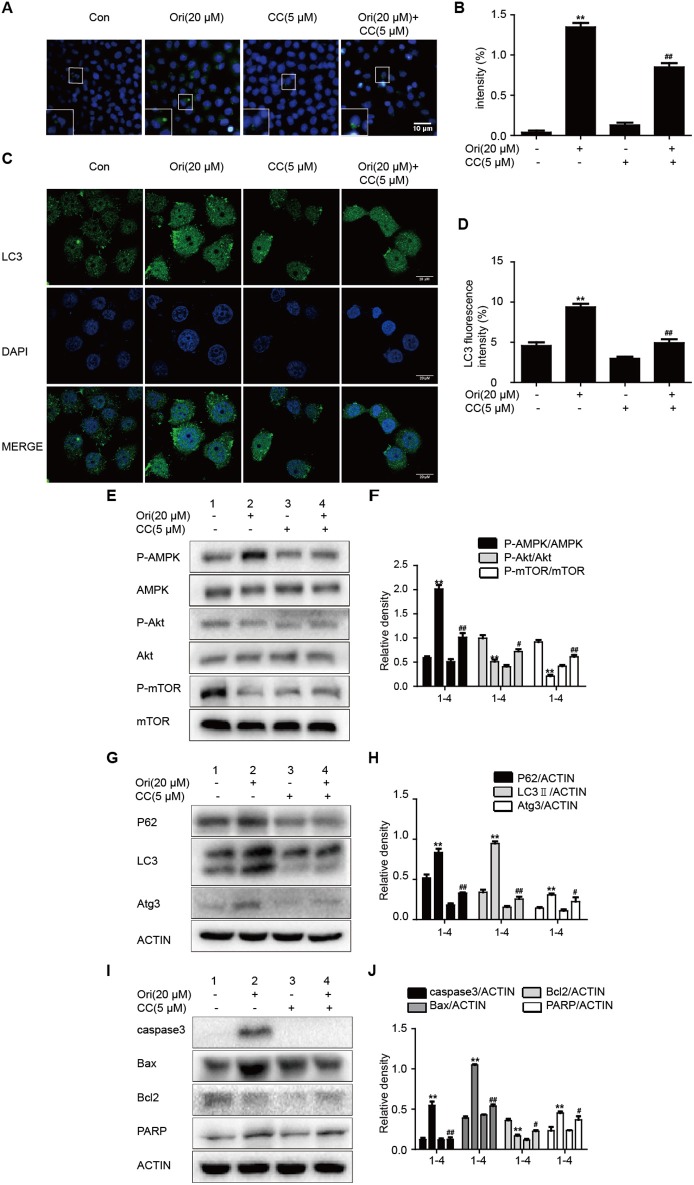
Ori initiates autophagy, apoptosis and Akt/mTOR signaling by the regulation of AMPK. Cells were treated with Ori (20 μM) in the absence or presence of compound c (5 μM) for 18 h. **(A,B)** Cells were stained with an autophagosome specific green reagent and examined by fluorescence microscope. The green fluorescence intensity was analyzed by Image J. Scale bars: 10 μM. **(C,D)** Cells were stained with LC3 and DAPI and images were captured with confocal microscope. The LC3 fluorescence intensity was analyzed by Image J. Scale bars: 20 μM. **(E–J)** Western blots and statistical results showed the protein levels of P-AMPK, AMPK, P-Akt, Akt, P-mTOR, mTOR, P62, LC3, Atg3, caspase3, Bax, Bcl2, and PARP. The results showed the average of three independent experiments. **p < 0.01 vs. the control group. ^#^*p* < 0.05 and ^*##*^*p* < 0.01 vs. the Ori group.

AMPK signaling acts as an upstream effector of autophagy and participates in oncotherapy. Based on Ori's protective effect on the above pathways, we explored the interrelation between them by the addition of compound c (AMPK inhibitor). The number of autophagosomes was counted and LC3 puncta formation was analyzed by immunofluorescence. Ori-induced autophagosomes and representative protein LC3 accumulation were reduced by compound c (Figures 2A–D). Then Ori-activated P62, LC3, Atg3, and apoptotic proteins (Bax, caspase3, PARP) were reduced by compound c (5 μM), while the low levels of Akt/mTOR and Bcl2 in Ori-treated cells were elevated (Figures 2E–J).

### Ori Induces Apoptosis Through Autophagy Initiation

**Figure 3:**
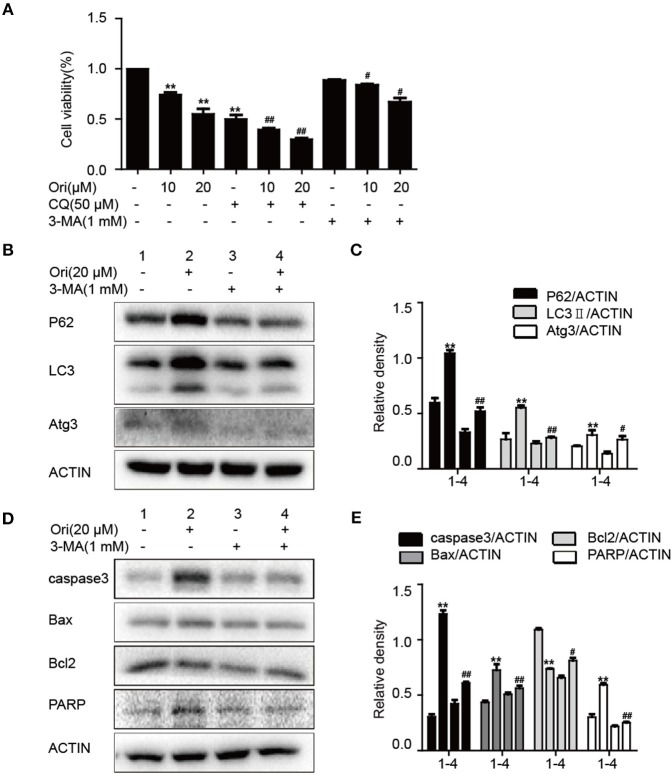
Ori induces apoptosis through autophagy initiation. **(A)** Cells were exposed to different concentrations (10 and 20 μM) of Ori either alone or in the presence of autophagy inhibitors (50 μM CQ and 1 mM 3-MA) for 18 h. Cell viability was calculated as the percentage relative to untreated cells which were considered as 100%. **(B–E)** Cells were seeded in 6-well plates and treated with Ori (20 μM) in the absence or presence of 3-MA (1 mM) for 18 h. Western blots and statistical results showed the protein levels of P62, LC3, Atg3, caspase3, Bax, Bcl2, and PARP. The results showed the average of three independent experiments. **p < 0.01 vs. the control group. ^#^*p* < 0.05 and ^*##*^*p* < 0.01 vs. the Ori group.

Pro-apoptotic molecules are widely used to enhance the regulation of autophagy in cancer treatment. So we added 3-MA (autophagy inhibitor) to examine the relationship between autophagy and apoptosis in Ori-treated cells. First, we examined the effects of 3-MA (1 mM) and CQ (50 μM) on Ori-induced cytotoxicity by CCK-8 assay (Figure 3A). The combination of CQ and Ori exerted better cytotoxic effects in A549 cells, while 3-MA antagonized the anti-tumor effects of Ori. These results indicated that autophagosomes induced by Ori possessed cell-killing effects in A549 cells. Then, Western blotting was used to detect the proteins related to apoptosis and autophagy (Figures 3B–E). Autophagy and apoptosis activated by Ori (20 μM) were restrained by 3-MA. Above all, Ori induced apoptosis by the AMPK/Akt/mTOR-dependent impaired autophagosomes accumulation.

### Ori Enhances Chemotherapeutic Sensitivity Through the Modulation of Autophagy and AMPK/Akt/mTOR Pathways

**Figure 4:**
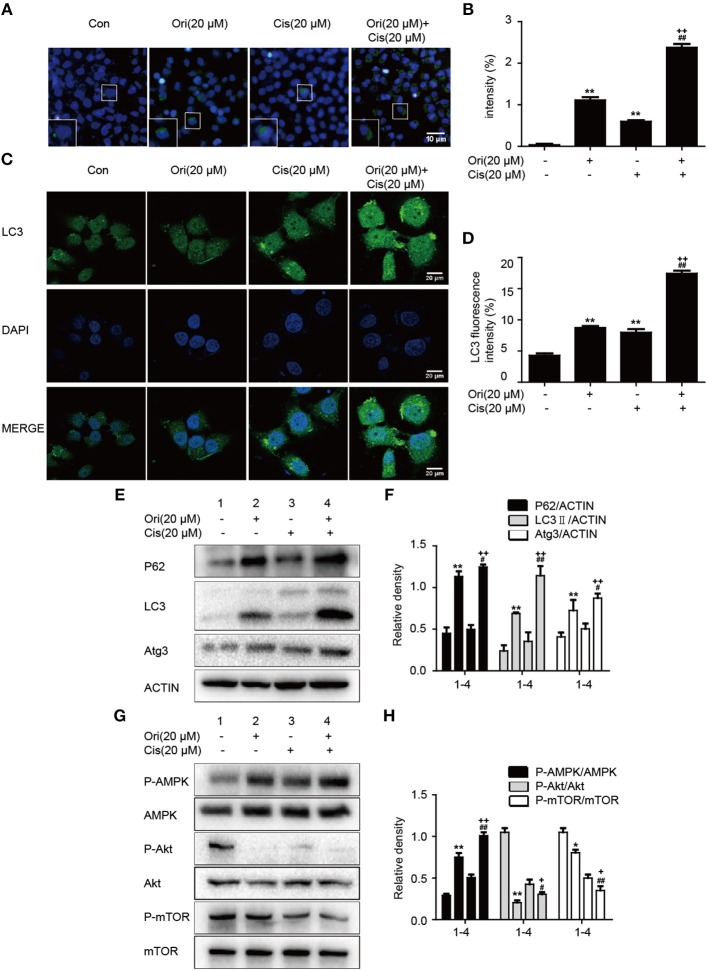
Ori enhances chemotherapeutic sensitivity through the modulation of autophagy and AMPK/Akt/mTOR pathways. A549 cells were exposed to Ori (20 μM) in the absence or presence of cisplatin (20 μM) for 18 h. **(A,B)** Cells were stained with an autophagosome specific green reagent and examined by fluorescence microscope. The green fluorescence intensity was analyzed by Image J. Scale bars: 10 μM. **(C,D)** Cells were stained with LC3 and DAPI and images were captured with confocal microscope. The LC3 fluorescence intensity was analyzed by Image J. Scale bars: 20 μM. **(E–H)** Western blots and statistical results showed the protein levels of P62, LC3, Atg3, P-AMPK, AMPK, P-Akt, Akt, P-mTOR, and mTOR. The results showed the average of three independent experiments. *p < 0.05 and **p < 0.01 vs. the control group. ^#^*p* < 0.05 and ^*##*^*p* < 0.01 vs. the Ori group. ^+^*p* < 0.05 and ^++^*p* < 0.01 vs. the Cis group.

**Figure 5:**
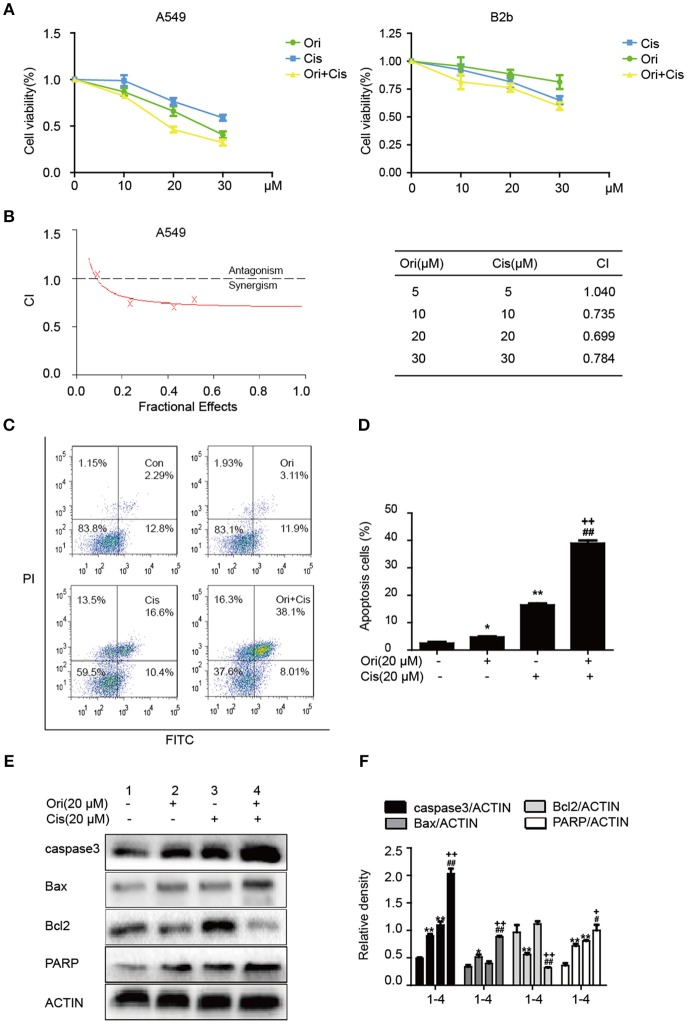
Ori enhances chemotherapeutic sensitivity through the activation of apoptosis. **(A)** A549 cells were treated with different doses (10, 20, and 30 μM) of Ori in the absence or presence of cisplatin (10, 20, and 30 μM) for 18 h. Cell viability was calculated as the percentage relative to untreated cells which were considered as 100%. **(B)** Data was added into CalcuSyn and combination index (CI) was analyzed in this experiment. CI <1 indicates synergism, while CI>1 indicates antagonism. A549 cells were exposed to Ori (20 μM) in the absence or presence of cisplatin (20 μM) for 18 h. **(C,D)** The percentages of apoptotic and necrotic cells were determined by flow cytometry. **(E,F)** Western blots and statistical results showed the protein levels of caspase3, Bax, Bcl2, and PARP. The results showed the average of three independent experiments. *p < 0.05 and **p < 0.01 vs. the control group. ^#^*p* < 0.05 and ^*##*^*p* < 0.01 vs. the Ori group. ^+^*p* < 0.05 and ^++^*p* < 0.01 vs. the Cis group.

Based on the results mentioned later (**Figures 5A,B**), we chose the corresponding doses and tested whether Ori sensitized cisplatin-induced cell death by the regulation of autophagy. Immunofluorescence and Western blotting were used to display impaired autophagosome accumulation. LC3 puncta and autophagosomes formation were also promoted in the group of Ori (20 μM) plus cisplatin (20 μM) (Figures 4A–D). P62, LC3, Atg3, and AMPK/Akt/mTOR expressions were more obvious in cotreatment with Ori and cisplatin (Figures 4E–H). Taken together, these results indicate that Ori increases cisplatin sensitivity through the role of autophagy and AMPK/Akt/mTOR.

### Ori Enhances Chemotherapeutic Sensitivity Through the Activation of Apoptosis

Chemotherapy resistance has always been an important factor in reducing the survival rate of cancer patients, so researchers investigate sensitizers to reverse the resistance. On the basis of anti-tumor potential of Ori, we further examined whether Ori could enhance cisplatin sensitivity by CCK-8 assay. Cisplatin (10, 20 μM) showed slight inhibition of tumor growth, while cell viability was evidently suppressed after the addition of Ori (10, 20 μM) (Figure 5A). Addtionally, 30 μM cisplatin showed cell-killing effects on B2b cells. Then, added these data into CalcuSyn and calculated the combination index according to the formula. CI <1 indicates synergism, while CI > 1 indicates antagonism. From our results, the combination of Ori (20 μM) and Cis (20 μM) showed the best synergism and these two doses were used in our experiments (Figure 5B). We further investigated the role of apoptosis in cisplatin plus Ori group, and the effect of Ori (20 μM) on apoptotic proteins (caspase3, Bax, Bcl2, and PARP) was more remarkable in cisplatin-treated cells (Figures 5E,F). Then we detected apoptosis activity by FASC and the results were consistent with related protein expression levels (Figures 5C,D). In summary, these results indicate that Ori possesses anti-tumor and cisplatin sensitizer potential by the activation of apoptosis, which was mediated by AMPK/Akt/mTOR-dependent autophagy initiation.

### Ori Alleviates Cisplatin-Induced Nephrotoxicity *in vivo*

**Figure 6:**
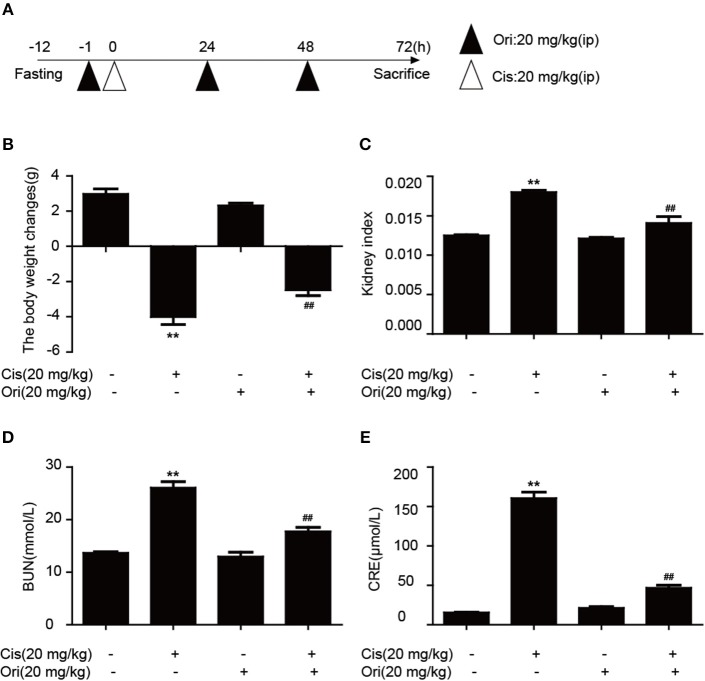
Ori alleviates cisplatin-induced nephrotoxicity *in vivo*. **(A)** Experimental design of Ori's therapeutic effects on cisplatin-induced kidney injury. After fasting for 12 h, intraperitoneal injection of cisplatin (20 mg/kg) was performed to establish an acute kidney injury model. Ori (20 mg/kg) was administered to the enterocoelia for 3 consecutive days. **(B,C)** Body and kidney weights were calculated and the kidney index was shown as kidney weight divided by body weight. **(D,E)** Blood was collected to detect the levels of BUN and CRE. The results showed the average of three independent experiments. **p < 0.01 vs. the control group. ^*##*^*p* < 0.01 vs. the Cis group.

According to the above results, we found the protective effect of Ori in cancer treatment and could enhance the sensitivity of cisplatin. While clinically recognized that with the use of chemotherapy drugs, nephrotoxicity induced by cisplatin also appears. So we chose a dose of 20 mg/kg cisplatin intraperitoneal injection for 3 days to induce acute kidney injury and Ori (20 mg/kg) injection simultaneously as a treatment (Figure 6A). We examined some indicators of kidney function and found that Ori could reduce cisplatin-induced high levels of BUN, CRE, kidney index, and weight loss (Figures 6B–E).

## Discussion

Ori, an ent-kaurane diterpenoid isolated from Isodon rubescens, functions as an anti-tumor compound. It has been demonstrated that Ori could regulate the levels of LC3II/I and some Atg proteins in A549 cells. However, Ori-regulated the levels of P62 in tumor growth and cisplatin resistance of lung cancer have not been investigated. P62 is critical protein to decide the process of autophagy flux. Generally, P62 will be degraded and autophagy flux is complete and fluent. Once the autophagy flux is blocked and the degradation of P62 will be also inhibited. Therefore, the levels of P62 and autophagosomes are vital in cancer treatment (27). In our study, Ori induced AMPK/Akt/mTOR-dependent autophagosome accumulation, which further activated apoptosis to inhibit lung cancer cell growth and increase cisplatin sensitivity. Moreover, Ori alleviated cisplatin-induced renal damage indexes (BUN, CRE and body weight).

Under stress conditions, autophagy exerts a pro-survival activity in carcinogenesis and drug resistance (29, 30). When autophagy occurs, cytoplasmic components (organelles, proteins) are wrapped by double-layer membrane vesicles and form autophagosomes, which are then transferred to lysosomes for degradation. This procedure aims to provide nutrients and energy for cellular activity. In these reactions, Atg genes and LC3 regulate the formation of autophagosome double-layer membranes. Under the participation of Atg3, LC3I is transformed into LC3II and tightly binds to the surface of autophagic vesicles, taking part in the extension of autophagosomes (31). As a vital tumor suppressor, P62 interacts with mTORC1 and negatively regulates lysosome acidification and autophagosome-lysosome fusion (32). Increasing evidence indicates that cisplatin can induce autophagy, and suppression of autophagy by 3-methyladenine(3-MA) strongly enhanced cisplatin sensitivity (21). Our results showed that Ori evidently inhibited cell growth and induced the over-expressions of LC3II, Atg3, P62 and the accumulation of autophagosomes under confocal and general fluorescence microscopy. The increased levels of LC3 and P62 tend to block autophagosomal maturation and degradation (suppression of autophagy at its late stage), therefore we combined Ori and CQ to examine the induced changes in autophagy flux. As expected, Ori induced increased expression of LC3II and acidic autophagosomes in cells treated with CQ. Thus, these results suggested that Ori inhibited autophagy flux and exerted an effect similar to that of CQ to cause the activation of autophagosomes (Figure 1). Then, the anti-tumor potential and autophagy initiation of Ori were weakened by the addition of 3-MA, so we considered that Ori-induced autophagosome accumulation caused lung cancer cell death (Figure 2). Based on the abovementioned results, we investigated the role of Ori in cisplatin-treated cells. Ori showed increased expression of LC3II, Atg3 and P62 and decreased cell viability after cotreatment with cisplatin, and we reached the conclusion that Ori increased cisplatin sensitivity by the inhibition of autophagy flux (Figure 4).

In the relevant pathways of cisplatin resistance, AMPK, Akt/mTOR and apoptosis also play an important role (18, 19, 33). AMPK is activated during situations in which the cellular level of ATP is decreased and therefore will inhibit tumor cell growth and enhance chemotherapy effect (17). For instance, the expression of AMPK-α was decreased in human breast cancer tissues and the AMPK activator metformin could have a positive impact on the effect of chemotherapy (34, 35). In addition, the Akt/mTOR pathway and apoptosis also participate in cisplatin resistance in various cancers. Recent studies indicate that inhibition of autophagy by andrographolide re-sensitizes cisplatin-resistant non-small cell lung carcinoma cells via activation of the Akt/mTOR pathways (36). While silencing long non-coding RNA ROR improves sensitivity of non-small-cell lung cancer to cisplatin resistance by inhibiting PI3K/Akt/mTOR signaling pathway (37). Additionally, it has been demonstrated that a prominent hallmark of platinum resistance is the evasion of apoptosis. Apoptotic signaling maintains a balance between cell death and cell survival, however, the dysfunction of pro- and anti-apoptotic proteins contributes to platinum resistance by reducing apoptosis. More evidence shows that agents activating apoptosis by targeting the Bcl-2 family, P53 and caspases can have a protective effect on sensitizing resistant cells (38–41). Therefore, we observed the effect of Ori on the above pathways and found that Ori enhanced the levels of AMPK, Bax, caspase3 and PARP and suppressed Akt, mTOR and Bcl2 expression in A549 cells (Figure 1). In cisplatin-treated cells, Ori had more obvious regulatory effects on apoptosis and Akt/mTOR (Figures 4, 5). Therefore, we considered that Ori increased the sensitivity of cisplatin by regulating apoptosis, AMPK and Akt/mTOR.

Research has shown that AMPK and autophagy frequently refer to the upstream regulators of apoptosis (17, 42). So we investigated the correlation among these pathways using compound c (AMPK inhibitor) and 3-MA (autophagy inhibitor). Results indicated that the overexpression of autophagy and apoptosis related proteins by Ori were reversed by 3-MA (Figure 3), and Ori-induced Akt/mTOR, autophagy and apoptosis-relevant changes were all impaired by compound c (Figure 2). In keeping with these results, LC3 puncta activated by Ori were reduced in the pretreatment of these two inhibitors. In summary, we could made conclusions that Ori induced apoptosis by AMPK/Akt/mTOR-dependent autophagosome accumulation and increased cisplatin resistance in NSCLC cells. In addition, with the use of chemotherapy drugs, cisplatin-induced nephrotoxicity is observed (43). However, the protective effect of Ori as anti-tumor agent should not be interfered by the side effect of cisplatin. So we constructed a renal injury mouse model induced by cisplatin and investigated whether Ori could have a therapeutic effect. As expected, Ori could alleviate cisplatin-induced high levels of BUN and CRE, weight loss and kidney index (Figure 6).

**Figure 7:**
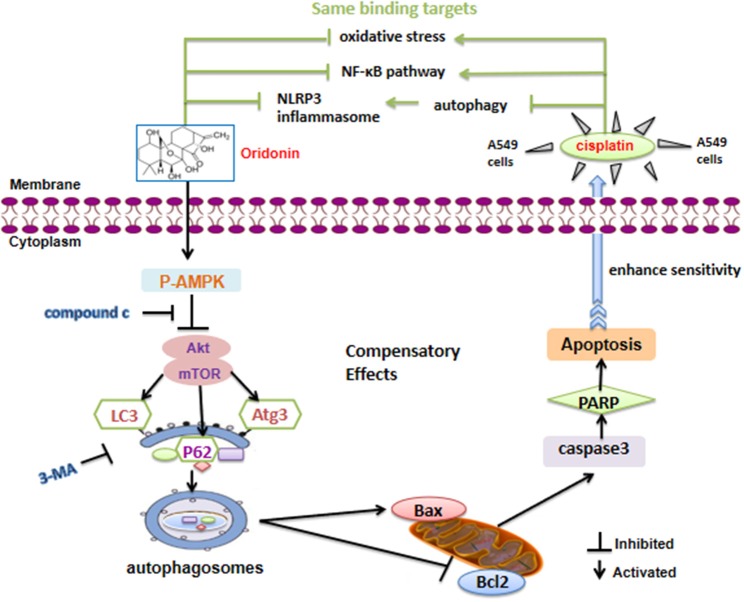
Schematic model of the increased cisplatin sensitivity by Ori in A549 cells. Ori induced apoptosis and enhanced the chemotherapeutic efficacy of cisplatin via AMPK/Akt/mTOR-dependent impaired autophagosome accumulation (black lines). And the same binding targets between Ori and Cis were also exhibited (green lines).

In summary, Ori displayed anti-tumor bioactivity and decreased cisplatin resistance by apoptotic signaling activation, which was modulated by AMPK/Akt/mTOR-dependent autophagy inhibition. Besides, Ori had a therapeutic effect on cisplatin-induced nephrotoxicity (Figure 7). Our results suggested that Ori, as a specific autophagy modulator, could be potentially developed as an adjuvant for further cancer treatment and chemotherapy resistance. Additionally, these findings highlighted the role of autophagosome activation and the related AMPK/Akt/mTOR pathways and apoptosis, thereby providing new ideas for the treatment of lung cancer.

## Data Availability

The raw data supporting the conclusions of this manuscript will be made available by the authors, without undue reservation, to any qualified researcher.

## Ethics Statement

All animal studies were reviewed and approved by the Animal Welfare and Research Ethics Committee of Jilin University.

## Author Contributions

HY, XC, and LP designed the research. HY, YG, XF, and XL performed the research. YG, XF, and XL helped to analyze the data. HY wrote the manuscript.

### Conflict of Interest Statement

The authors declare that the research was conducted in the absence of any commercial or financial relationships that could be construed as a potential conflict of interest.
